# Asymptomatic Bacterial Vaginosis Is Associated With Depletion of Mature Superficial Cells Shed From the Vaginal Epithelium

**DOI:** 10.3389/fcimb.2020.00106

**Published:** 2020-03-10

**Authors:** D. Elizabeth O'Hanlon, Pawel Gajer, Rebecca M. Brotman, Jacques Ravel

**Affiliations:** ^1^Institute for Genome Sciences, University of Maryland School of Medicine, Baltimore, MD, United States; ^2^Department of Microbiology and Immunology, University of Maryland School of Medicine, Baltimore, MD, United States; ^3^Department of Epidemiology and Public Health, University of Maryland School of Medicine, Baltimore, MD, United States

**Keywords:** women's health, gynecology, BV, vaginal microbiome, vaginal microbiota

## Abstract

Previous studies have described bacterial vaginosis (BV) as associated with increased cell-shedding from the cervicovaginal epithelium. Cell-shedding in excess of cell-proliferation is thought to decrease epithelial barrier function and increase susceptibility to infection. This study evaluated the number of shed cells in mid-vaginal smears from women with a diagnosis of symptomatic BV (sBV, *n* = 17), asymptomatic BV (aBV, *n* = 71), or no BV (*n* = 104) by Amsel criteria. The sBV smears contained significantly more shed cells (median 158/100X field) than no BV smears (median 91/100X field), *p* = 7.2e−9. However, we observed that aBV smears contained significantly fewer shed cells (median 35/100X field) than no BV smears, *p* = 22.0e−16. The sizes of cell-aggregates (cells shed in sometimes multilayered sections with intact cell-cell attachments) followed the same pattern. Cell-aggregates in sBV smears were significantly larger (median ~220,000 μm^2^) than those in no BV smears (median ~50,000 μm^2^), *p* = 1.8e−6, but cell-aggregates in aBV smears were significantly smaller (median ~7,000 μm^2^) than those in no BV smears, *p* = 0.0028. We also compared the superficial cell index (SCI), a measure of cervicovaginal epithelial cell maturity, in no BV and aBV smears with relatively low numbers of shed cells (≤50/100X field). The SCI of no BV smears was significantly higher (median 0.86) than that of aBV smears (median 0.35), *p* = 4.3e−98, suggesting a depletion of mature cells with exposure and shedding of underlying immature cells in aBV with low number of shed cells. These results indicate that aBV may contribute disproportionately to the increased susceptibility to reproductive tract infections associated with BV. Our findings remained true when considering only those smears in which the microbiota comprised a diverse set of strict and facultative anaerobic bacteria [Community State Type IV (*n* = 162)], thus excluding those dominated by *Lactobacillus* spp. This is consistent with our developing hypothesis that high-shedding sBV and low-shedding aBV could be temporally separated phases of the same condition, rather than two separate forms of BV. These findings might inform future work on clinical management of symptomatic and asymptomatic bacterial vaginosis.

## Introduction

The cervicovaginal microbiota is known to contribute to a woman's protection against and susceptibility to reproductive tract infections (RTIs). Women whose cervicovaginal microbiota is composed predominantly of *Lactobacillus* spp. are at decreased risk of many sexually transmitted infections, including HIV (Taha et al., 1998; Gosmann et al., 2017), HPV (Mitra et al., 2016), HSV-2 (Cherpes et al., 2003), trichomoniasis (Brotman et al., 2012), gonorrhea and chlamydia (Wiesenfeld et al., 2003) compared to women with microbiota comprising strict and facultative anaerobic bacteria. The latter is what broadly characterizes bacterial vaginosis (BV), a condition that is defined differently in clinical and research settings (Mckinnon et al., 2019). Clinically, Amsel-BV (Amsel et al., 1983) is characterized by the presence of certain clinical signs with patients reporting symptoms (symptomatic BV [sBV]) or no symptoms (asymptomatic BV [aBV]) on direct questioning (Fleury, 1983; Eschenbach et al., 1988). In research studies, Nugent-BV is characterized, through microscopic examination of a vaginal smear, by a predominance of Gram-negative *Gardnerella* and *Bacteroides* spp. morphotypes rather than Gram-positive *Lactobacillus* spp. morphotypes (Nugent et al., 1991). Vaginal microbiota assessment using culture-independent approaches based on the quantification and characterization of bacterial 16S rRNA gene sequences defines molecular-BV when a microbiota both lacks high relative abundance of *Lactobacillus* spp. and is composed predominantly of a wide array of strict and facultative anaerobic bacteria [Community State Type (CST) IV] (Gajer et al., 2012). Neither Nugent-BV nor molecular-BV can distinguish between sBV and aBV in the absence of a clinical examination and patient questioning. Although the concordance among the methods is not complete, it is generally true that Amsel-BV, Nugent-BV, and molecular-BV are progressively more inclusive: in a given population, more women are positive for Nugent-BV than for Amsel-BV, and more are positive for molecular-BV than for Nugent-BV (Mckinnon et al., 2019).

The cervicovaginal epithelium is the site at which multiple defenses against STIs and RTIs are deployed (Hickey et al., 2011) and *Lactobacillus* spp. contribute to: 1/ physical defenses, including mucus-trapping of pathogens (Nunn et al., 2015), suppression of inflammatory de-keratinization of epithelial cells (Zárate et al., 2009) and control of cell proliferation often necessary for infection (Edwards et al., 2019); 2/ immunological defenses, including suppression of pro-inflammatory signaling through toll-like receptors (Mirmonsef et al., 2011) and suppression of pro-inflammatory cytokine expression (Jespers et al., 2017); and 3/ biochemical defenses, including inducing expression of protective peptides (Yarbrough et al., 2015) and production of lactic acid (Tachedjian et al., 2017).

The homeostatic balance between the proliferation/maturation and shedding/loss of cells on the vaginal epithelium is likely critical in maintaining these defenses. Balanced proliferation/maturation and shedding/loss means the luminal surface consists of dead detaching cells and the adherence of pathogens to these sloughing cells has been hypothesized as a mechanism to provide some protection to the underlying living cells, which are vulnerable to productive infection (Anderson et al., 2014). Accelerated shedding/loss of cells from the vaginal epithelium, therefore, could be expected to decrease this protective function and thus increase susceptibility to STIs and RTIs. The composition of the cervicovaginal microbiota may affect the proliferation/maturation and shedding/loss balance: approximately twice as many shed epithelial cells were found in a study of vaginal smears from women with Nugent-BV, vs. women with predominantly *Lactobacillus* spp. microbiota (Amegashie et al., 2017). Additionally, the composition of the cervicovaginal microbiota has been shown to control the expression of the host microRNA miR-193b which reduces cell proliferation (Edwards et al., 2019). Cell proliferation was lower in women with a BV-associated microbiota (Edwards et al., 2019), as were host proteins associated with epithelial maturation (Zevin et al., 2016). Further supporting this finding are observations of fewer mature and more immature epithelial cells in cervicovaginal samples from women lacking lactobacilli (Fowler, 2012). At the same time, increased cell-shedding could eliminate non-*Lactobacillus* spp. bacteria attaching to the epithelium, such as *G. vaginalis* or *Atopobium vaginae*, which can form resilient biofilms (Hardy et al., 2016). Only symptomatic Amsel-BV is typically treated (Workowski and Bolan, 2015); however, inflammatory changes on the cervicovaginal epithelium are found in women with Nugent-BV (Thurman et al., 2015; Jespers et al., 2017) and molecular-BV (Anahtar et al., 2015), and so it is not surprising that the increased susceptibility to infections associated with BV also accrues to women with Nugent-BV (Cherpes et al., 2003; Wiesenfeld et al., 2003) and molecular-BV (Brotman et al., 2012, 2014; Gosmann et al., 2017).

The objective of the current study was to determine whether the number and maturity of shed epithelial cells observed in sBV and aBV differ, utilizing a collection of cervicovaginal smears for which microbiota composition and clinical data were available.

## Materials and Methods

### Clinical Samples

The current study utilized samples and data from the UMB-HMP study (Ravel et al., 2013). A total of 135 non-pregnant, reproductive-aged women took part in a 10-weeks observational longitudinal study during which participants self-collected daily vaginal samples, under a protocol approved by the Institutional Review Boards of the University of Alabama at Birmingham and of the University of Maryland School of Medicine. Written informed consent was obtained from all participants.

The study included examination by a clinician at enrollment, week 5 and 10, or at interim times if vaginal symptoms were reported. None of the samples used in this study were positive for STI or yeast infection. The clinician's record for each examination included a diagnostic of BV performed according to the Amsel criteria (Amsel et al., 1983). A diagnosis of symptomatic BV (sBV) was established when the participant reported symptoms on direct questioning and fulfilled at least three of the four Amsel's criteria; a diagnosis of asymptomatic BV (aBV) was established when the participant did not report symptoms but fulfilled at least three of the Amsel's criteria. Each day, participants self-collected a vaginal swab, smeared it on a microscope slide and placed the slide in a protective sleeve. Participants dropped off the slides to the clinic every week. The slides were Gram-stained and scored for Nugent-BV (Nugent et al., 1991) by three independent readers (Ravel et al., 2013). Another daily swab stored in Amies transport medium and immediately frozen at −20°C was used for extraction and purification of genomic DNA using a QIAsymphony robotic platform and QIAGEN CellFree 500 kits (QIAGEN, Valencia CA); Metataxonomic analysis (Marchesi and Ravel, 2015): the V3-V4 hypervariable regions of bacterial 16S rRNA genes were amplified and sequenced on an Illumina MiSeq Instrument to obtain the bacterial composition and abundance of each sample as described previously (Fadrosh et al., 2014). Sequencing was performed at the Institute for Genome Sciences' Genomic Resource Center (GRC) at the University of Maryland School of Medicine (marylandgenomics.com). CST (Gajer et al., 2012) were assigned using VALENCIA, a novel nearest centroid classification algorithm based on the classification of over 13,000 vaginal microbiota dataset. This approach to CST assignment is more robust than standard within-study hierarchical clustering and allows for between-studies comparisons (Ravel, 2018).

### Microscopy

Vaginal smears collected on the day of clinical examination (study entry, week 5 and 10 for each participants) were evaluated as described below and the findings evaluated with respect to associated clinical and microbiota data.

**Figure 1:**
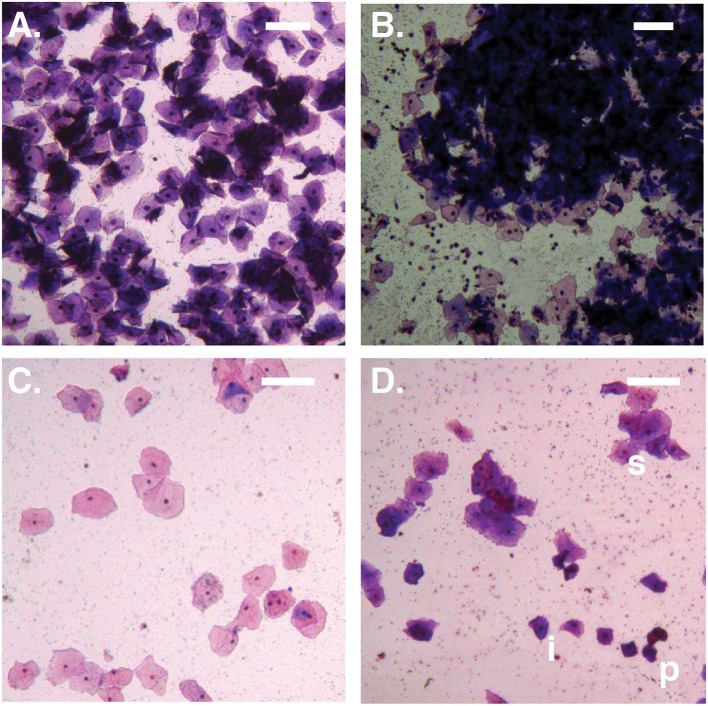
Bright-field micrographs (100X total magnification). **(A)** No Amsel-BV sample with cell count >100/100X field, showing predominantly superficial cells shed as singletons. **(B)** Symptomatic Amsel-BV sample with cell count >100/100X field, showing predominantly superficial cells shed in large aggregates. **(C)** No Amsel-BV sample with cell count <50/100X field, showing predominantly superficial cells. **(D)** Asymptomatic Amsel-BV sample with cell count <50/100X, showing superficial cells (example marked s) shed in combination with intermediate (example marked i) and parabasal (example marked p) cells. Scale bars represent 100 μm.

A total of 192 smears from 126 women met the criteria above and were available to be examined. Following the method previously described to evaluate cervicovaginal epithelial cell-shedding (Amegashie et al., 2017), slides were visualized using a Zeiss Plan-ACHROMAT 10x objective on a Zeiss Primo Star microscope (Carl Zeiss Microscopy LLC, Thornwood NY) for a total magnification of 100x, and images of three “representative” fields were captured from each slide using a Zeiss Axiocam ICc3 camera and software. “Representative” fields were located within the main body of the smear, not near the start, end, or margins; “representative” fields were chosen to exclude aggregations of epithelial cells too dense to distinguish cells. All complete epithelial cells in the three images were counted (i.e., cells falling partly outside the image were not counted) and the mean count calculated. Images of three “representative” cell-aggregations were captured, also from locations within the main body of the smear, away from the start, end, or margins. Cell-aggregation size was measured using the “measure > outline” tool of the imaging software (unit μm^2^) (AxioVision v 4.8.2.0); the mean cell-aggregation size was calculated. When the mean epithelial cell count was ≤50, the three captured images were further analyzed, counting the number of superficial cells (i.e., cells with a condensed nucleus, large cytoplasmic space, and polygonal shape with thin, angular margins; Anderson et al., 2014). From this, the superficial cell index (SCI = number of superficial cells/number of all epithelial cells) was calculated (range 0.0–1.0; Stupnicki and Teter, 1970). A SCI of 1.0 corresponds to high numbers of mature cells and low or no immature cells, while a low SCI corresponds to low numbers of mature epithelial cells and high numbers of immature epithelial cells. Example of different types of smears are shown on Figure 1. All measurements are listed in Supplementary Table 1.

### Statistical Analysis

Cell counts, cell-aggregate sizes, and SCI values are reported as median and interquartile range. Comparisons of cell counts were performed by fitting a Bayesian Laplace subject-wise random effects model to the log_10_ transformed data. Comparisons of cell-aggregate sizes were made in the same way. SCI values were computed within a Bayesian binomial mixed effects model with subject-wise random intercept. Adjustment for multiple testing was performed using false discovery rate (FDR). For all comparisons, exact *p*-values are reported. Within woman pairwise comparison of log_10_ cell counts and aggregation sizes was made using a Bayesian Poisson within subject two group comparison model. All scripts used in this study are available on GitHub at https://github.com/ravel-lab/BV_CELL_SHEDDING.

## Results

### Comparison of Total Epithelial Cell Counts Between Asymptomatic, Symptomatic and No BV Smears

**Figure 2:**
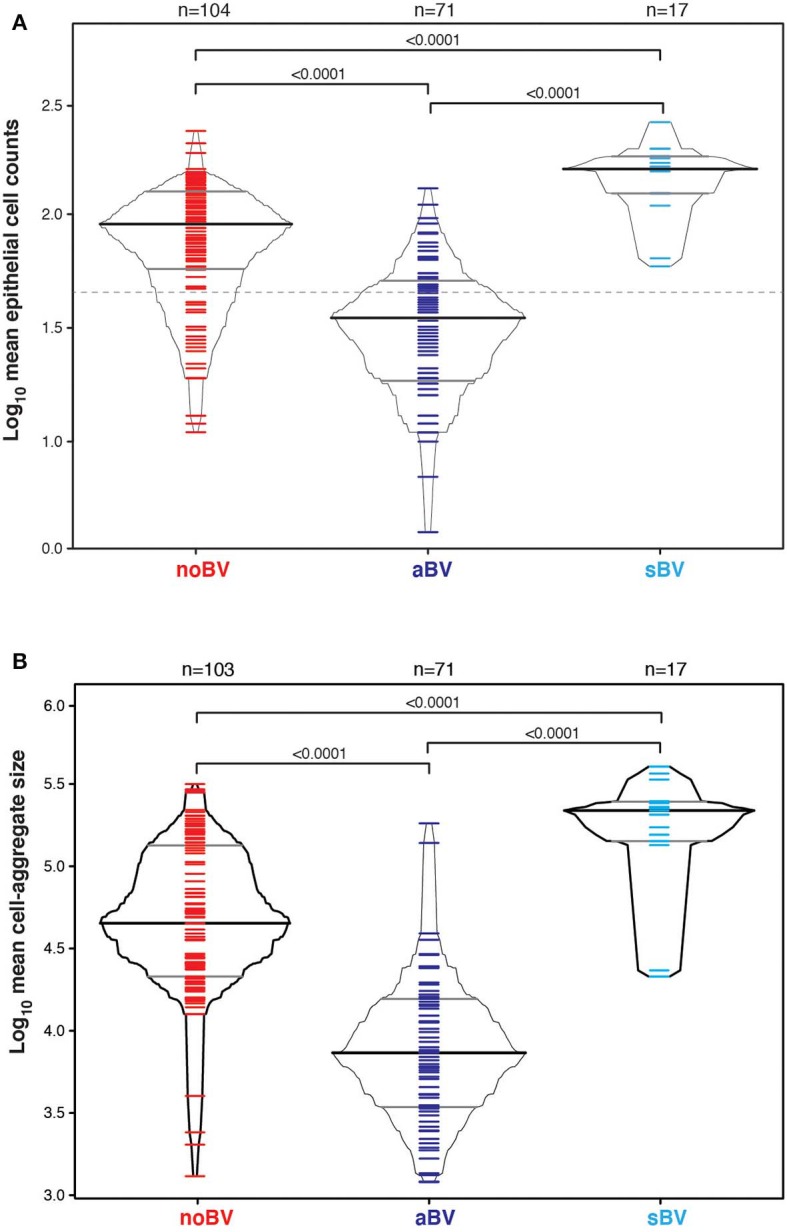
**(A)** Box-percentile plot of log_10_ mean epithelial cell counts in different Amsel diagnostic groups. Dash line indicates 50 mean epithelial cells. **(B)** Box-percentile plot of log_10_ mean cell-aggregate sizes in different diagnostic groups. The median, 25 and 75th percentiles are marked with line segments across each box. Numbers at the top axis indicate the number of samples in each group. Ticks within percentile-boxes show individual sample values.

Cell counts were first considered based on diagnostic group only, without reference to microbiota CST information. The median cell count of samples with no diagnosis of Amsel-BV (noBV, *n* = 104) was 91/100X field (interquartile range 58–126). This was significantly lower than the median cell count for samples with a diagnosis of symptomatic Amsel-BV (sBV, *n* = 17), which had median 158/100X field (IQR 124–179), *p* = 7.2e−09. Additionally, the median cell count of noBV samples was significantly higher than that of samples with a diagnosis of asymptomatic Amsel-BV (aBV, *n* = 71), which had a median 35/100X field (IQR 19–50), *p* = 2.4e−16 (Figure 2A). In addition, median cell counts of sBV and aBV samples were significantly different, *p* < 10e−12.

### Comparison of Cell-Aggregates Size Between Asymptomatic, Symptomatic and No BV Smears

**Figure 3:**
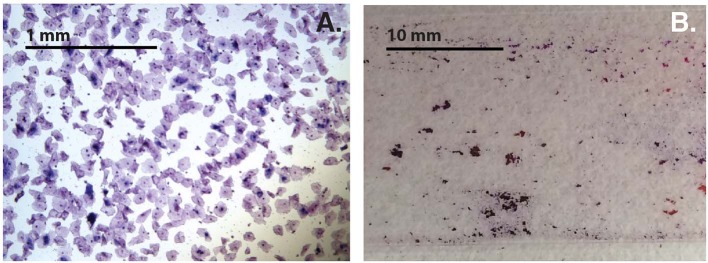
**(A)** Bright-field micrograph (100X total magnification) showing sample with single cells and small cell-aggregates. **(B)** Photograph without magnification showing samples with cell-aggregates large enough to see with the naked eye.

Variability of cell-aggregates on the slides was apparent. Some slides had evenly dispersed cells with aggregates of only a few cells each (Figure 3A), while other slides had multiple aggregations so extensive and dense that they could be distinguished without magnification (Figure 3B). Cell-aggregate size measurements indicated that in noBV samples the median cell-aggregate size (50,000 μm^2^, IQR 20,000–140,000 μm^2^) was significantly smaller than that for sBV samples, which had a median cell-aggregate size of 220,000 μm^2^ (IQR ~140,000–240,000 μm^2^), *p* = 1.6e−14. Additionally, the median cell-aggregate size of noBV samples was significantly larger than that of aBV samples, which had a median cell-aggregate size of ~7,000 μm^2^ (IQR 3,000–14,000 μm^2^), *p* < 2.22e−16 (Figure 2B). Lastly, the median cell-aggregate sizes of sBV and aBV samples were significantly different, *p* = 2.2e−55.

### Comparison of the Superficial Cell Index Between Asymptomatic, Symptomatic and No BV Smears

**Figure 4:**
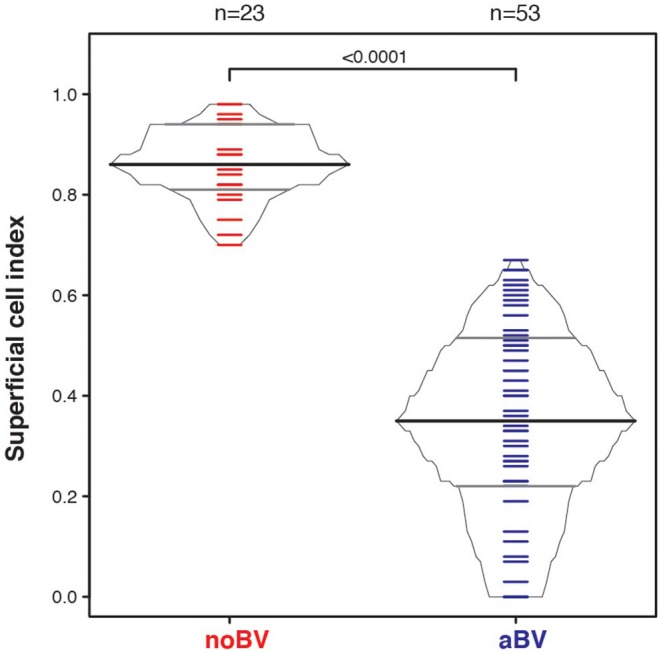
Box-percentile plot of superficial cell index in no Amsel-BV and asymptomatic Amsel-BV samples with a mean epithelial cell counts of <50.

The superficial cell index (SCI) was calculated for samples with a mean cell count of ≤50/100X field, corresponding to a total of 78 samples from 54 women. Mean cell count of ≤50/100X field was chosen since it included almost all aBV samples and was well-represented among noBV samples. The median SCI of noBV samples (*n* = 23) was 0.86 (IQR 0.81–0.94), significantly higher than that of aBV samples (*n* = 53), which had a median SCI of 0.35 (IQR 0.22–0.51), *p* = 4.3e−98 (Figure 4). No sBV samples had mean cell count ≤50/100X field.

### Comparison of Total Epithelial Cell Counts, Cell-Aggregate Sizes and SCI Stratified by Amsel Diagnostic Groups and CSTs

**Figure 5:**
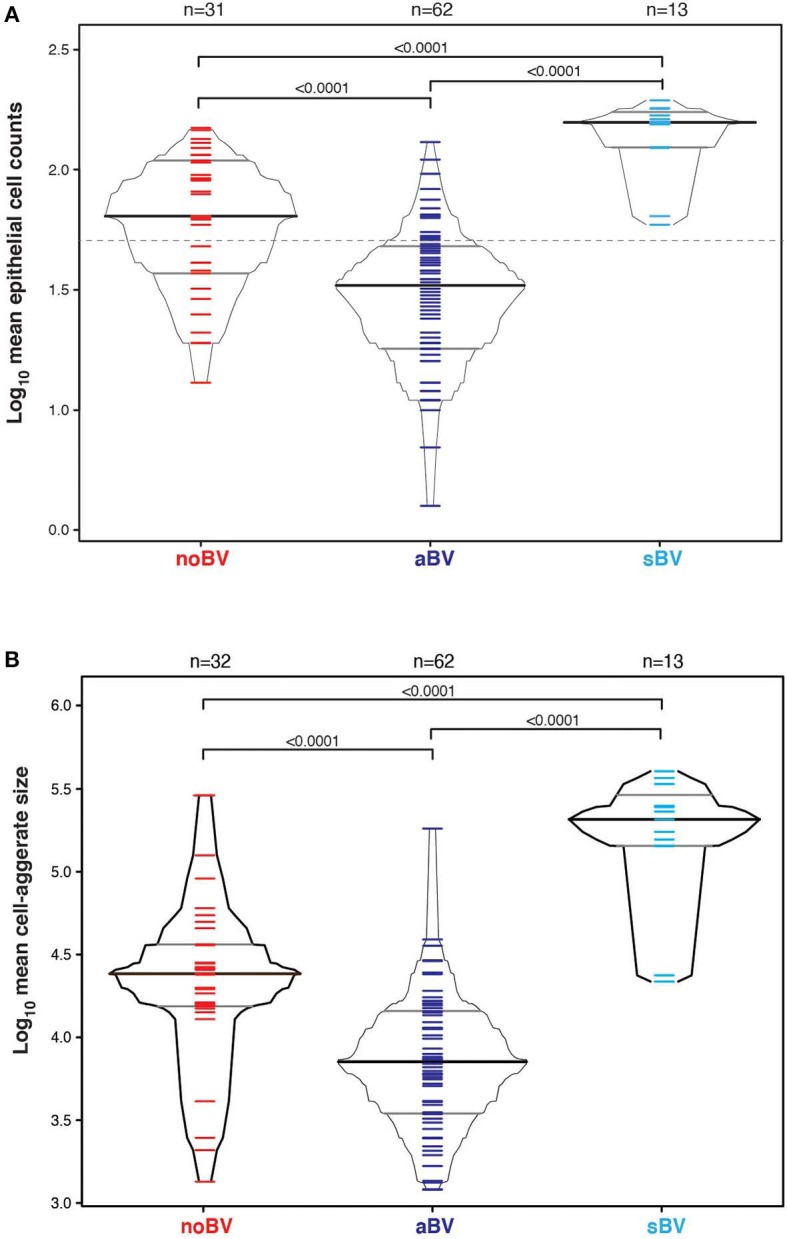
**(A)** Box-percentile plot of log_10_ mean epithelial cell counts in different diagnostic groups for only CST IV samples. Dash line indicates 50 mean epithelial cells. **(B)** Box-percentile plot of log_10_ mean cell-aggregate sizes in different diagnostic groups for only CST IV samples. The median, 25 and 75th percentiles are marked with line segments across each box. Numbers at the top axis indicate the number of samples in each group. Ticks within percentile-boxes show individual sample values.

**Figure 6:**
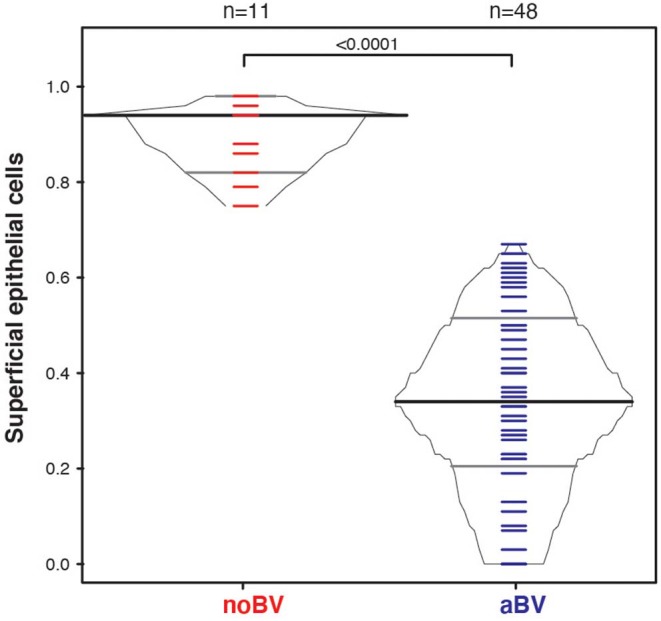
Box-percentile plot of superficial cell index in no Amsel-BV and asymptomatic Amsel-BV samples for only CST IV samples with a mean epithelial cell counts of <50.

Interestingly, differences between noBV, sBV, and aBV remained when samples were considered based on Amsel diagnostic groups and CSTs. Among samples assigned to CST IV (microbiota comprising a wide range of facultative and strict anaerobic bacteria and a lack of *Lactobacillus* spp.), the median cell count of noBV samples (64/100X field, IQR 38–112), *n* = 31, Amsel-BV negative, but molecular-BV positive was significantly lower than that of sBV samples (157/100X field, IQR 157–171, *n* = 13), *p* = 9.5e−7, and significantly higher than aBV samples (33/100X field, IQR 18–48), *n* = 62), *p* = 0.00011 (Figure 5A). In addition, median cell counts of sBV and aBV samples assigned to CST IV were significantly different, *p* = 1.3e−24. Similarly, the median cell-aggregate size of CST IV noBV samples (20,000 μm^2^, IQR 15,000–25,000 μm^2^) was significantly smaller than that of CST IV sBV samples (25,000 μm^2^, IQR 15,000–36,000 μm^2^), *p* = 5.8e−16, and significantly larger than that of aBV samples (7,000 μm^2^, IQR 3,600–13,000 μm^2^), *p* = 1.06e−9 (Figure 5B). Further, the median cell-aggregate sizes of sBV and aBV samples assigned to CST IV were significantly different, *p* = 3.15e−45. Further, Lastly, among the 61 samples with mean cell count of ≤50/100X field and that were CST IV, the median SCI of noBV samples (0.94 IQR 0.84–0.95), *n* = 11) was significantly higher than that of aBV samples (0.34, IQR 0.22–0.51, *n* = 48), *p* = 5.3e−73 (Figure 6). There were too few samples with a microbiota dominated by *Lactobacillus* spp. that were categorized as sBV (*n* = 4, all CST III) or aBV (*n* = 4, one CST I and three CST III) to permit any analysis for CSTs other than CST IV.

## Discussion

The equilibrium among the continuous processes of cell proliferation, maturation and shedding on the vaginal epithelium is probably essential to maintaining an effective barrier against pathogenic and non-pathogenic microbes alike. Here, we report an association of symptomatic Amsel-BV (sBV) with evidence of increased cell-shedding, and of asymptomatic Amsel-BV (aBV) with both decreased cell-shedding and increased presence of immature epithelial cells.

**Figure 7:**
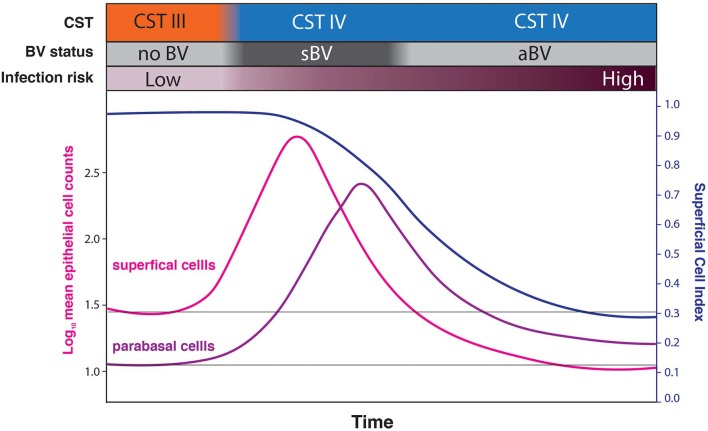
Conceptual model hypothesizing that sBV may be rapidly followed by aBV. Under this hypothesis, the increased cell-shedding events associated with sBV exhaust the superficial and intermediate epithelial cell layers resulting in reduced cell-shedding, and exposure and loss of immature epithelial cells (low SCI) associated with aBV. This progression is hypothesized to be associated with increased risk of infections.

These findings raise two intriguing possibilities. Firstly, rather than sBV and aBV being two forms of the condition, we hypothesize that they are sometimes two phases of a single form. Under this model (Figure 7), sBV may be rapidly followed by aBV. Though the dataset presented here is effectively cross-sectional and cannot directly address this possibility (there were only 7 women who provided both sBV and aBV samples, collected several weeks apart). Under this hypothesis, the increased cell-shedding events associated with sBV exhaust the superficial and intermediate epithelial cell layers resulting in reduced cell-shedding, and exposure and loss of immature epithelial cells associated with aBV. This hypothesis is supported by the finding that there are no significant differences in microbiota structure between aBV and sBV [high frequency of CST IV (93.94 and 76.47%, respectively, *p* = 0.205) and CST III (4.55 and 23.53%, respectively, *p* = 0.096)] (Supplementary Figure 1), although absolute abundances of bacteria have not been evaluated and could be different between sBV and aBV. We are currently undertaking larger, longitudinal studies to address these possibilities.

Secondly and more importantly, our findings suggest that aBV might be associated with more severe disruption of the vaginal epithelium than sBV, and hence lead to a greater increase in susceptibility to infections. By analogy, the spermicide nonoxynol-9 (Hillier et al., 2005) is known to cause accelerated cell-shedding (Niruthisard et al., 1991), thinning (Vincent et al., 2011), disruption (Hoffman et al., 2004) and pro-inflammatory changes (Smith-Mccune et al., 2015) on the vaginal epithelium, and was found to increase women's susceptibility to HIV (Wilkinson et al., 2002), as well as susceptibility to HSV-2 (Cone et al., 2006) and HPV (Roberts et al., 2007) in a mouse model. Since aBV is associated with similar physiological effects on the epithelium, we hypothesize it would be associated with an increased risk of infections.

If validated, the hypothesis that aBV does have a particularly disruptive effect on the cervicovaginal epithelium would make it necessary to reevaluate the relative importance of sBV and aBV. While vaginal symptoms have been linked in part to a patient's self-report (Klebanoff et al., 2004) thus symptoms perception, six women in this study presented with sBV and aBV at separate clinical visits over the course of the study (Supplementary Table 1), indicating that women who have had aBV also recognize sBV. However, we hypothesize that based on our findings, there may be relevant biological differences between aBV and sBV. Current CDC guidelines recommend antibiotic treatment only for sBV, not aBV (Workowski and Bolan, 2015). Future work may lead clinicians to reconsider the merits of treating aBV and amending counseling messages for women with aBV to manage their increased susceptibility to infection, for example by avoiding unprotected sexual intercourse. However, expanded screening based on high-resolution molecular assays of both the microbiota and the state of the epithelium would need to be implemented at routine gynecological visits, and more importantly, the development of specific therapies to address the epithelial disruption associated with aBV would be needed.

In our study, the median cell count of noBV samples assigned to CST IV (70/100X field, *n* = 32) was significantly lower than that of other noBV samples (93/100X field, *n* = 69), *p* = 0.0384 (Supplementary Figure 2). This is likely because molecular-BV broadly defines BV and is not limited to Amsel-sBV and Amsel-aBV (as evidenced by our data set, in which 31/104 noBV samples were CST IV). Interestingly, it has been noted that the BV-associated increased risk of HIV acquisition is higher in studies using molecular-BV (defined as all forms of CST IV) as the exposure variable, compared to studies using Amsel-BV or Nugent-BV (Mckinnon et al., 2019). A recent meta-analysis showed that Amsel-BV was associated with a 2-fold increase in risk for HIV acquisition (OR: 1.93, 95% CI: 1.45–2.57; Atashili et al., 2008). A subsequent study suggested that molecular-BV was associated with a 4-fold hazard ratios compared to *L. crispatus*-dominated samples (HR:4.41, 95% CI: 1.17–16.61, *p* = 0.028; Gosmann et al., 2017). Molecular-BV is associated with proinflammatory cytokines (Gosmann et al., 2017) and activated HIV-target cells (Anahtar et al., 2015) in the cervicovaginal epithelium and thus with increased risk of HIV acquisition. These findings support a higher risk associated with asymptomatic molecular-BV, and suggest mechanisms involving impaired physiological state of the epithelium and increased pro-inflammatory markers. The above epidemiologic studies of BV and risk for HIV suggest that molecular-BV might trend toward higher point estimates for HIV risk than Amsel-BV because molecular-BV includes more women with at-risk aBV states. To our knowledge, no studies have directly assessed the risk of HIV or other STI acquisition in women with molecular-BV vs. sBV; further studies are needed to test our hypothesis.

## Data Availability Statement

Raw counts and metadata used in this study are available in Supplementary Table 1.

## Ethics Statement

Samples used in this study were archived vaginal smears obtained in accordance with protocols approved by the University of Maryland Baltimore Institutional Review Board.

## Author Contributions

DO'H, RB, and JR designed the study. DO'H executed the laboratory part of the study. PG performed the statistical analyses. DO'H, PG, RB, and JR wrote/edited the manuscript.

### Conflict of Interest

JR is co-founder of LUCA Biologics, a biotechnology company focusing on translating microbiome research into live biotherapeutics drugs for women's health. The remaining authors declare that the research was conducted in the absence of any commercial or financial relationships that could be construed as a potential conflict of interest.
