# Supplementary figures and images for: Asymptomatic Bacterial Vaginosis Is Associated With Depletion of Mature Superficial Cells Shed From the Vaginal Epithelium
Source: Front Cell Infect Microbiol. 2020 Mar 10;10:106. doi: 10.3389/fcimb.2020.00106 (PMC7076050; doi:10.3389/fcimb.2020.00106)

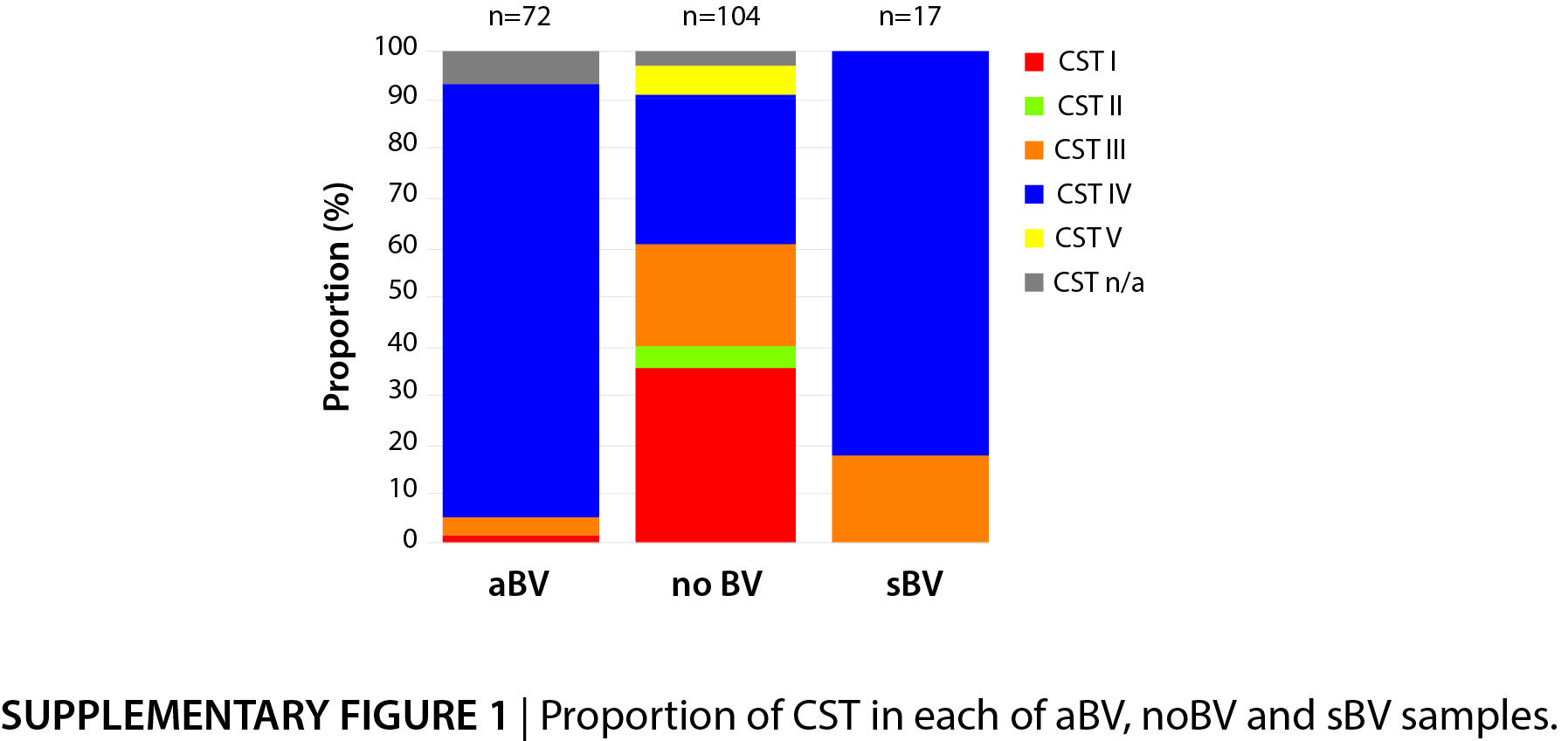

Supplement: Supplementary file 1 [file Image_1.jpg]

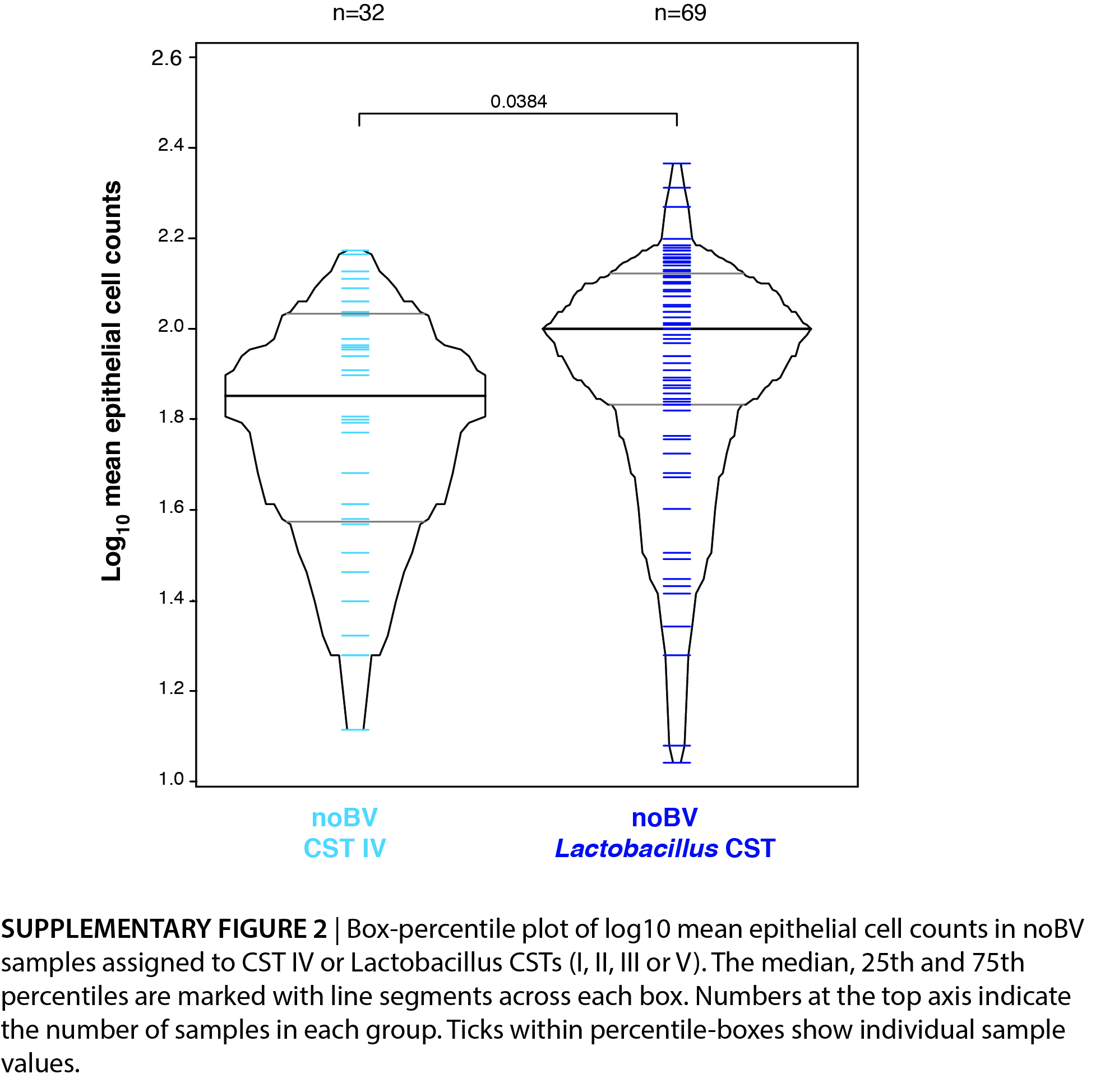

Supplement: Supplementary file 2 [file Image_2.jpeg]
